# Relative Hypocalcaemia and Muscle Cramps in Patients Receiving Imatinib for Gastrointestinal Stromal Tumour

**DOI:** 10.1155/SRCM/2006/48948

**Published:** 2006

**Authors:** Jamal M. Zekri, Martin H. Robinson, Penella J. Woll

**Affiliations:** Weston Park Hospital, University of Sheffield, Sheffield S10 2SJ, UK

## Abstract

*Purpose*. Imatinib treatment causes muscle cramps in up to 40% of patients, but their pathogenesis is unknown. We present a case series illustrating an association between imatinib, relative hypocalcaemia, and the development of cramps. *Patients*. The index patient developed muscle spasms and cramps after receiving imatinib for gastrointestinal stromal tumour (GIST) for 5 months. The adjusted serum calcium had dropped to the lower limit of normal. The low serum calcium and muscle cramps improved on stopping imatinib and recurred on rechallenge. We reviewed the medical records of 16 further patients. *Results*. Two patients reported muscle cramps (12%). There was a rapid and sustained reduction in adjusted serum calcium in the first 6 months from 2.45 ± 0.11 mmol/L (mean ± SD) to 2.30 ± 0.08 mmol/L (*p* = 0.025). *Conclusion*. Imatinib treatment of GIST is associated with reduction in serum calcium which may explain the development of neuromuscular symptoms. In patients receiving imatinib, serum electrolytes should be monitored and muscle cramps treated by correction of serum calcium, or an empirical trial of quinine sulphate.

## INTRODUCTION

Imatinib mesylate is a tyrosine kinase inhibitor targeted to BCR-ABL, PDGFR, and KIT. It has unprecedented activity in chronic myelogenous leukaemia (CML) and gastrointestinal stromal tumours (GISTs), and has dramatically changed the clinical management of these tumour types [1–4]. GISTs characteristically have an activating mutation in the KIT receptor. Objective response rates to imatinib in GIST are just over 50% [5, 6]. Recent data suggest that the presence of exon 11 mutations in KIT predict for response to imatinib, with a response rate of 83% in this patient group [7]. In patients with advanced GIST, imatinib treatment has improved median survival from less than 1 year to more than 3 years, with 65% of patients free of progression and 85% alive at one year [6]. Imatinib is administered by mouth as a daily dose of 400–800 mg until tumour progression.

The adverse effects of imatinib are mostly mild and manageable. The most common adverse effects reported by GIST patients are listed in Table 1. Musculoskeletal effects of imatinib are reported in 25% of GIST patients and 20–40% of CML patients, including arthralgia, myalgia, and muscle cramps, but are rarely dose-limiting [2, 5, 6, 8]. The pathophysiology of these effects is uncertain. Here, we report a patient in whom the development of cramps and involuntary movements while receiving imatinib was associated with a significant reduction in adjusted serum calcium levels. We then studied the calcium level changes in a cohort of patients receiving imatinib for GIST and found that reduction in serum calcium occurred in all.

## CASE REPORT

A 38-year-old woman with extensive abdominal GIST commenced imatinib mesylate (Glivec, Novartis) 400 mg/day in October 2003. Her adjusted serum calcium was 2.54 mmol/L (normal range 2.2–2.55). Imatinib was well tolerated, and her tumour mass slowly responded to treatment. She was active (performance status 1), had a normal diet and no evidence of malabsorption. During the fifth month of therapy, she complained of increasing muscle twitches and cramps, locked fingers, and spasm of the tongue. Her adjusted serum calcium was 2.28 and magnesium 0.75 (0.7–0.95) mmol/L, both at the lower limit of the normal ranges.

**Figure 1:**
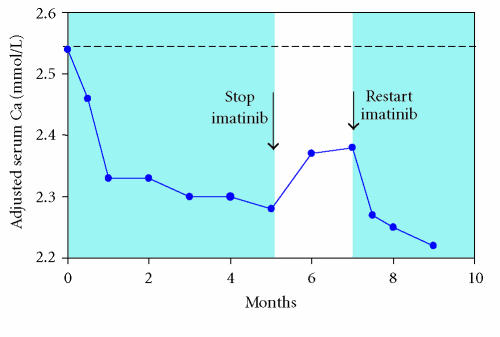
Changes in adjusted serum calcium in the index patient from the start of imatinib treatment. Reference range 2.2–2.55 mmol/L.

On stopping imatinib, the symptoms rapidly resolved, and the serum calcium and magnesium recovered to 2.37 and 1.0 mmol/L respectively (Figure 1). Imatinib was restarted after 3 weeks, with oral calcium and magnesium supplements, but the cramps recurred. She was then given quinine sulphate 300 mg/day, with complete resolution of the cramps. Fifteen months later, she continues on imatinib 400 mg/day with quinine sulphate 300 mg/day. The tumour mass continues to regress. Her serum calcium remains at the lower limit of the normal range, but her cramps are controlled with quinine sulphate.

## REVIEW OF CASE SERIES

In view of the findings in this patient, we undertook to review the medical notes of a cohort of 17 consecutive patients treated in our institution with imatinib for GIST. We noted the occurrence of musculoskeletal adverse effects and recorded adjusted serum calcium levels. Magnesium levels had not been consistently assessed and hence are not available for this report. Results are expressed as mean ± standard deviation, and two-tailed paired *t* tests were used to test the difference in means.

The characteristics of the 17 patients are shown in Table 2. These are comparable to those in reported trial series. All had inoperable, locally advanced or metastatic KIT-positive GIST. None was receiving bisphosphonates. The median progression-free and overall survival had not been reached at time of analysis. In addition to the index patient, only one other reported symptoms of involuntary movements and cramps. These did not lead to imatinib dose reduction or withdrawal, but were managed with quinine sulphate 300 mg prn.

**Figure 2:**
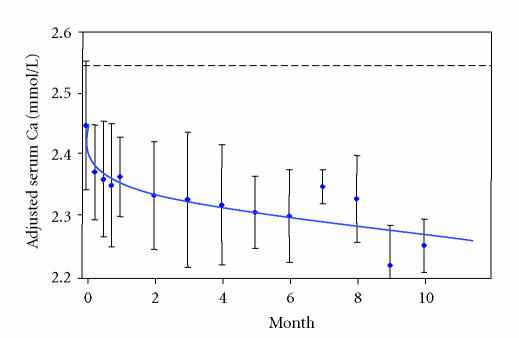
Changes in adjusted serum calcium in 17 patients receiving imatinib for advanced GIST, shown as mean ± standard deviation. Reference range 2.2–2.55 mmol/L.

At the start of imatinib treatment, all patients had normal serum calcium levels (mean 2.45 ± SD 0.11 mmol/L). All patients exhibited a rapid and sustained fall in adjusted serum calcium during treatment with imatinib to 2.30 ± 0.08 mmol/L at 6 months (Figure 2) although few readings were below the lower limit of the normal range. The reduction in adjusted serum calcium was statistically significant at each time point from week 2 to month 6 (*P* = 0.002 − 0.05).

## DISCUSSION

The appearance of neuromuscular symptoms in the index patient after 4 months of imatinib, their resolution on stopping the drug and reappearance on restarting it are highly suggestive of a causal effect of imatinib. As muscle cramps occur in up to 40% of patients on receiving imatinib, this was not an unexpected finding [2, 5, 6, 8]. However, the clear association with adjusted serum calcium levels, seen here, suggested that the reduced calcium level was also an effect of imatinib treatment, and possibly associated with the muscle cramps. To explore this association further, we studied a further 16 patients receiving imatinib for GIST. Interestingly, we found that imatinib treatment is consistently associated with a rapid and sustained fall in adjusted serum calcium, albeit usually within the normal reference range. We therefore hypothesise that imatinib treatment is associated with relative hypocalcaemia that can precipitate neuromuscular symptoms in some patients.

Muscle cramps and other neuromuscular or symptoms have been widely reported in patients receiving imatinib. They usually occur in the hands, feet, calves, and thighs, and may be tetanic in nature [2]. The cramps tend not to change over time with respect to pattern, frequency, and severity. They do tend to have consistent triggers, and some patients report experiencing them mainly at night or with exertion. Although such patients do not typically have levels of ionized calcium or magnesium below the lower limit of normal, some benefit from calcium and magnesium supplements [2, 9]. Oral fluids have been encouraged, and quinine sulphate has been used empirically, with improvement in some patients. Recent data suggest that GIST can progress rapidly when imatinib is discontinued [10], so this cannot be recommended as a strategy for dealing with cramps unless they are extremely severe.

Interestingly, a reduction in the serum calcium has previously been noted in patients receiving imatinib for CML, but is not widely recognised. Steegmann et al [11] found significant reductions in serum calcium and phosphate in the patients receiving the imatinib for interferon-resistant or -intolerant CML, but no change in serum creatinine or creatinine clearance. There was no association with neuromuscular symptoms in this study. The failure to observe an association between imatinib and relative hypocalcaemia in other large series is probably because the changes are typically within the normal reference range. The high reported incidence of muscle cramps however suggests that relative hypocalcaemia may be a common problem in these patients.

The pathophysiological basis for this association remains uncertain but some possibilities can be considered. Firstly, although KIT is expressed on renal tubular cells, their functions there are unknown. Imatinib could therefore be exerting a direct effect on renal tubular KIT receptors, resulting in relative hypocalcaemia. Secondly, imatinib is a member of a family of protein tyrosine kinase inhibitors which can induce marked changes in cell excitability and ion homeostasis. Indeed, imatinib blocks low voltage-activated T-type calcium channels in human embryonic kidney cells [12]. Thus imatinib could have a nonspecific effect on calcium homeostatsis that is not KIT receptor-mediated.

Our findings suggest that imatinib treatment in GIST patients is commonly associated with relative hypocalcaemia, which may contribute to the occurrence of neuromuscular symptoms in up to 40% of patients. The adjusted serum calcium rarely falls below the lower limit of normal. It may be prolonged, but it is not cumulative. We recommend that serum electrolytes should be monitored in these patients, so that symptomatic hypocalcaemia can be corrected. In the presence of muscle cramps, electrolyte replacement or quinine sulphate can be tried. Imatinib should not be discontinued unless the cramps are very severe.

## Figures and Tables

**Table 1:** Incidence of common adverse effects (%) in patients receiving imatinib 400 mg/day for GIST, among 470 patients in a randomised clinical trial (from Verweij et al [6]).

Adverse effect	Grades 1/2	Grades 3/4

Anaemia	82	7
Oedema	69	3
Fatigue	62	6
Pleuritic pain	47	4
Nausea	46	2.5
Diarrhea	46	1.7
Cramps	37	1.3
Granulocytopenia	34	7
Rash	24	2.3
Myalgia	24	0.2
Arthralgia	13	0

**Table 2:** Patient characteristics.

Age (years)	Median 60, range 38–83
Sex	Male 7, female 10
Starting dose of imatinib	400 mg, 12 patients
	800 mg, 5 patients
Duration of treatment, months	Median 23, range 3–39
